# Pertussis vaccination status and vaccine acceptance among medical students: multicenter study in Germany and Hungary

**DOI:** 10.1186/s12889-019-6516-8

**Published:** 2019-02-12

**Authors:** Mandy Böhme, Karen Voigt, Erika Balogh, Antje Bergmann, Ferenc Horváth, Joachim Kugler, Jörg Schelling, Jeannine Schübel, Henna Riemenschneider

**Affiliations:** 1 Department of General Practice, Medical Clinic III, University Hospital Carl Gustav Carus, Technische Universität Dresden, Fetscherstr. 74, 01307 Dresden, Germany; 2 Department of Public Health Medicine, University of Pécs Medical School, Sziget út 12, Pécs, 7624 Hungary; 3 Department of Public Health, Faculty of Medicine, Semmelweis University Budapest, Nagyvárad tér 4, Budapest, 1089 Hungary; 4 Department of Health Sciences /Public Health, Medical Faculty, Technische Universität Dresden, Fetscherstr. 74, 01307 Dresden, Germany; 5 Department of General Practice and Family Medicine, Ludwig-Maximilians-Universität, München, Pettenkoferstr. 8a, 80336 Munich, Germany

**Keywords:** Pertussis, Vaccination, Medical students, Cross-sectional study

## Abstract

**Background:**

Medical students are at risk of contracting and transmitting infectious diseases such as pertussis. Complete vaccination status is important to protect own, patient and public health. Knowing own vaccination status is elementary for following current vaccination recommendations, including boosters. We aimed to assess pertussis vaccination status and vaccination acceptance among medical students of different nationalities.

**Methods:**

A cross-sectional multicenter health survey at German and Hungarian universities enclosed international medical students in the 1st, 3rd and 5th year of study. Self-reported data from 2655 students regarding pertussis vaccination status were analyzed. Subgroup analysis enclosed data of German (*n* = 1217), Hungarian (*n* = 960) and other nationality (*n* = 478) students (“other”).

**Results:**

More Hungarians reported basic immunization (39.0% vs 15.8% Germans vs 24.3% others, *p* ≤ 0.05). Booster vaccination was reported more by Germans (60.5% vs 43.6% Hungarians vs 36.0% others, *p* ≤ 0.05). Germans were more likely to report being unvaccinated (3.7% vs 0.9% Hungarians, *p* ≤ 0.05). More medical students of other nationalities were unaware of their pertussis vaccination status (37.4% vs 20.0% Germans/ 16.5% Hungarians, *p* ≤ 0.05). 75.2% (*n* = 1931) rated pertussis vaccinations as absolutely necessary (86.2% Hungarians vs 69.8% Germans/ 66.1% others, *p* ≤ 0.05).

**Conclusions:**

Positive attitudes towards vaccinations were reported but a large group reported insufficient vaccination status and being not aware of their status, especially among international students. Hungarians possibly have a better vaccination status than reported, based on mandatory vaccinations in childhood. The low awareness of vaccination status has implications for future booster vaccinations. All students should be informed about current recommendations and receive vaccination offers in frames of low-threshold medical services.

## Background

Pertussis is a highly contagious bacterial disease which can be prevented by vaccination. Since the introduction of the pertussis vaccine in the 1950s, the incidence and mortality rate have decreased drastically, especially in the industrial world. However, pertussis still poses a big threat to public health. According to the World Health Organization (WHO), in 2013, more than 160,000 people were verifiable infected with pertussis worldwide and around 63,000 children under the age of five died from the disease [1, 2]. In regions with high vaccination rates (≥90%), such as major parts of Europe, the United States and Canada, the number of pertussis infections has been increasing again [3, 4]. Based on the data from 28 of 31 European Economic Area (EEA) member states with national surveillance systems, 9.1 pertussis cases per 100,000 inhabitants were reported in EEA 2014. However, there are regional differences: While Germany reported more than 12,000 cases (15.3 cases per 100,000 inhabitants) in 2014, in Hungary only 20 cases were reported that year (0.2 cases per 100,000 inhabitants) [4]. Even though pertussis is often described as "children’s disease", the incidence is currently increasing also in adolescents and adults. Since among these groups the disease often passes in a mild form or even asymptomatic and not verified by diagnostic tests, the reported incidence rates can be underestimated. Undetected infection can be transmitted to vulnerable unvaccinated populations that have a higher risk for severe complications [4].

According to the WHO, the vaccination against pertussis is part of the routine vaccinations in the general population [1]. Vaccination policies are put into practice in the various European nations very differently [5]. In many countries, such as Germany, France and the Netherlands, vaccination regulations are distributed to the public as recommendations [6]. As an example, in Germany the basic immunization involves four recommended vaccinations at the age of 2, 3, 4 and 11–14 months as well as two booster doses at the age of 5–6 and 9–17 years. Adults should receive a vaccine combined with a pertussis component, when their next tetanus booster dose is due or if they have close contact with newborn babies (“cocooning strategy”). Those working in the healthcare sector are advised to get a booster, unless they had a pertussis vaccination in the past 10 years [7, 8]. In some European countries, such as Hungary, Slovenia and Finland, the vaccination against the majority of vaccine-preventable diseases is mandatory [6]. In Hungary, the mandatory pertussis vaccination for children schedules three vaccinations at the age of 2, 3 and 4 months, and three booster doses at the age of 18 months, 6 years and since 2009 at the age of 11 years [9, 10]. Pertussis booster vaccinations for adults were introduced in Hungary a few years ago. These non-obligatory recommendations include booster doses based on “cocooning strategy” and pregnant women in endemic areas, and – since 2015 – the dTap (diphtheria, tetanus, pertussis) combination booster vaccination every 10 years for adults [11–13]. No specific recommendations for healthcare workers exist in Hungary [6].

Immunization history of people working in healthcare, including medical students in practice is of great importance, since they have a higher risk of infection and they pose a potential transmitting danger to the patients [14, 15]. Also, due to the high mobility of students (as a result of advancing Europeanisation processes i.e. 2262 Germans studied medicine/health science in Hungary in 2015/2016 [16, 17]), and encounters of people from different countries with different vaccination regulations, the risk for infectious diseases increases. Nevertheless, previous studies from Germany, France and Switzerland have demonstrated insufficient pertussis vaccination numbers of 17.1 to 72.7% in nursing and medical students [18–23].

This study aims to present the self-reported pertussis vaccination status of medical students of German, Hungarian and other nationalities studying in Germany (where vaccinations are recommended) and Hungary (where vaccinations are mandatory). The paper intends to gain knowledge about vaccination gaps, potential risk groups, and the assessment of the importance of the pertussis vaccination by people with medical insight. Moreover, the vaccination status of students in various phases of their studies is to be examined. The focus will be on German students both in Germany and Hungary, where they represent a major part of foreign students.

## Methods

### Study design and survey instrument

This cross-sectional multicenter study was conducted in collaboration with Technische Universität Dresden and Ludwig-Maximilians-Universität Munich in Germany as well as Semmelweis University Budapest and University of Pécs in Hungary. The health survey questionnaire was developed in a multiple Delphi process carried out by all collaborative partners. It includes questions regarding aspects of health behavior and risk behavior, including vaccination status (no vaccination; basic immunization without booster; basic immunization with booster; don’t know) and acceptance (assessed importance absolutely necessary; partly necessary; mostly necessary; unnecessary/dangerous). The questionnaire is based on validated instruments (e.g. SF-36 [24]) and previous surveys of Technische Universität Dresden and Semmelweis University [25–27]. The paper-pen based 9-page questionnaire was developed and consented in English, and was then translated to German and Hungarian. The feasibility of the questionnaires in all three languages was tested in pretests at the study sites in February 2014 in all languages (*n* = 131). Minor revisions were done to optimize the reliability of the questionnaires in all languages. Ethics approval was received for all study sites.

### Study participants and setting

1st, 3rd and 5th academic year medical students were invited to participate in the study voluntarily and anonymously during mandatory seminars/tutorials and lectures. The study purpose and the consent of participants were declared. The data collection took place in 2014 at all study centers (Dresden, Munich, Budapest and Pécs), targeting about 5000 registered students in total.

In addition to German and Hungarian students, we enclosed international students in Hungary (who represent about 40% of students at medical faculties in Hungary).

### Data management and statistical analysis

The data were recorded anonymously and no personal data was collected. The recorded data is stored at the Department of General Practice/TU Dresden in accordance with the applicable data protection regulations. Only authorized persons have access to the data. The data analysis was performed using SPSS 22.0. Pearson’s chi^2^-tests and z-tests (Bonferroni adjusted in case of comparison of more than 2 subgroups) were used to determine whether there were significant differences between frequencies regarding different subgroups. Due to the high variety of students‘backgrounds (85 different nationalities), three categories were created for the variable “nationality”: German, Hungarian and “other”. The categories German and Hungarian only include single citizenships; cases of dual citizenships are included in “other”. As part of a further analysis, the pertussis vaccination status was examined among the subgroup of Germans studying in Germany and in Hungary.

In order to show differences in gender distribution the binomial test was performed. Based on nonparametric distribution of the data Bonferroni adjusted Mann-Whitney-tests and Welch-test were used for comparing means of metric data (e.g. age) of the different subgroups. Logistic regressions were executed to meet the complexity of influencing factors (based on bivariate analysis) on complete vaccination status as well as to control effects of intercorrelation. It was examined, how a complete vaccination status is influenced by variables such as place of study, year of study, nationality, and the subjective assessment of the necessity of being vaccinated against pertussis. A complete vaccination status was only applied to students who stated to have received both the basic immunization and a booster dose (regardless the point in time and type of vaccine) not those with no or insufficient immunization. The answer “do not know” was not considered for the regression (*n* = 794). Since the pertussis vaccination is to be assessed as absolutely vital for all medical staff, the variable “importance of vaccination” was divided in two categories: “absolutely necessary” and “not absolutely necessary” (“partly necessary”, “mostly unnecessary”, “unnecessary/dangerous”).

## Results

### Student population

Of about 5000 registered students in 2014, 2961 students completed the survey questionnaire. After data check, this study includes data of a total of 2816 medical students in their first, third and fifth year of study. One thousand sixty-two students participated in Germany (589 in Dresden, 473 in Munich), and 1754 students took part in Hungary (770 in Pécs, 984 in Budapest). Of these, 2677 students (95.1%) stated their pertussis vaccination status, and 2655 (94.3%) added information about their nationality. Table 1 shows a summary of sociodemographic characteristics.

**Table 1:** Sociodemographic characteristics of medical students (*n* = 2655)

Characteristics		Nationality			
		German	Hungarian	Other	Total
Age*	mean (y)	22.9	21.6	23.5	22.5
	SD	±3.6	±2.5	±3.5	±3.3
Gender^†^	male (n)	37.9% (458)	36.2% (346)	40.4% (192)	37.7% (996)
	female (n)	62.1% (750)	63.8% (611)	59.6% (283)	62.3% (1644)
	ratio (m/f)	0.61	0.57	0.68	0.61
University	Dresden (n)	95.0% (535)	0,0% (0)	5.0% (28)	563
	Munich (n)	87.1% (385)	0,0% (0)	12.9% (57)	442
	Pécs (n)	24.4% (181)	51.5% (382)	24.1% (179)	742
	Budapest (n)	12.8% (116)	63.7% (578)	23.6% (214)	908
	All (n)	45.8% (1217)	36.2% (960)	18.0% (478)	2655
Study year	1st	49.4% (601)	42.1% (404)	34.1% (163)	44.0% (1168)
	3rd	29.4% (358)	30.5% (293)	39.3% (188)	31.6% (839)
	5th	211.2% (258)	27.4% (263)	26.6% (127)	24.4% (648)

*p ≤ 0.05 (Welch-test)

^†^Missings *n* = 15

Averaging 23.5 years of age, students of other nationalities were significantly older than students from Germany and Hungary. There were considerably more women (62.3%) than men among the students (binomial test; *p* ≤ 0.001). Among the students studying in Germany the majority had German nationality (91.5%; *n* = 920), none had Hungarian nationality, and the remaining students had a different or dual citizenship 8.5% (*n* = 85). In Hungary, 58.2% of the students were of Hungarian nationality (*n* = 960; 51.5% Pécs vs 63.7% Budapest), 18.0% (*n* = 297) were of German nationality, and 23.8% (*n* = 393) were of other nationality (Table 1).

### Self-reported vaccination status of medical students of German, Hungarian and other nationalities

Complete vaccination status regarding pertussis (basic and booster vaccination) was reported by 50.0% of the medical students in the total sample, while 25.7% reported having received only the basic immunization and 2.4% reported being not vaccinated. A further 21.9% did not know what their pertussis vaccination status was (Table 2). There were significant differences between the nationalities (χ^2^ = 250.5/ df = 6/ *p* ≤ 0.001) and the places of study (χ^2^ = 216.2/ df = 9/ *p* ≤ 0.001) (Table 2) as well as the years of study (χ^2^ = 247.3/ df = 6/ *p* ≤ 0.001).

**Table 2:** Self-reported vaccination status regarding pertussis among medical students in Dresden, Munich, Pécs and Budapest by nationality, n = 2655

		Self-reported vaccination status regarding pertussis; % (n)			
University	Nationality (n)	No vaccination	Basic immunization, without booster	Basic immunization, with booster	Don’t know
Dresden	German (535)	1.3%_a_ (7)	12.3%_a_ (66)	70.8%_a_ (379)	15.5%_a_ (83)
	Hungarian (0)	0.0% (0)	0.0% (0)	0.0% (0)	0.0% (0)
	Other (28)	3.6%_a_ (1)	17.9%_a_ (5)	50.0%_b_ (14)	28.6%_a_ (8)
	All(563)	1.4% (8)	12.6% (71)	69.8% (393)	16.2% (91)
Munich	German (385)	6.8%_a_ (26)	17.7%_a_ (68)	57.1%_a_ (220)	18.4%_a_ (71)
	Hungarian (0)	0.0% (0)	0.0% (0)	0.0% (0)	0.0% (0)
	Other (57)	1.8%_a_ (1)	14.0%_a_ (8)	54.4%_a_ (31)	29.8%_b_ (17)
	All (442)	6.1% (27)	17.2% (76)	56.8% (251)	19.9% (88)
Pécs	German (181)	2.8%_a_ (5)	21.0%_a_ (38)	43.1%_a_ (78)	33.1%_a_ (60)
	Hungarian (382)	1.6%_a_ (6)	35.3%_b_ (135)	46.1%_a_ (176)	17.0%_b_ (65)
	Other (179)	5.0%_b_ (9)	22.3%_a_ (40)	25.1%_b_ (45)	47.5%_c_ (85)
	All (742)	2.7% (20)	28.7% (213)	40.3% (299)	28.3% (210)
Budapest	German (116)	6.0%_a_ (7)	17.2%_a_ (20)	50.9%_a_ (59)	25.9%_a_ (30)
	Hungarian (578)	0.5%_b_ (3)	41.3%_b_ (239)	42.0%_a_ (243)	16.1%_b_ (93)
	Other (214)	0.0%_b_ (0)	29.4%_c_ (63)	38.3%_a_ (82)	32.2%_a_ (69)
	All (908)	1.1%(10)	35.5% (322)	42.3% (384)	21.1% (192)
Total	German (1217)	3.7%_a_ (45)	15.8%_a_ (192)	60.5%_a_ (736)	20.0%_a_ (244)
	Hungarian (960)	0.9%_b_ (9)	39.0%_b_ (374)	43.6%_b_ (419)	16.5%_a_ (158)
	Other (478)	2.3%_a,b_ (11)	24.3%_c_ (116)	36.0%_c_ (172)	37.4%_b_ (179)
	All (2655)	2.4% (65)	25.7% (682)	50.0% (1327)	21.9% (581)

_a,b,c_Each letter specifies a subset of ‘Nationality’ categories whose column shares are not significantly different at the 0.05 level (Z-test)

Mainly German students reported to be completely vaccinated (60.5% vs 43.6% Hungarian vs 36.0% other, *p* ≤ 0.05). The percentage of students reporting a basic immunization was highest among the Hungarians (39.0% vs 15.8% German vs 24.3% other, *p* ≤ 0.05). The percentage of students not having any immunization at all was significantly higher among Germans, compared to Hungarian students (3.7% vs 0.9%, *p* ≤ 0.05). The number of students not knowing about their vaccination status was highest among students of other nationalities (37.4% vs 20.0% German vs 16.5% Hungarian, *p* ≤ 0.05). Table 2 shows the self-reported pertussis vaccination status of medical students at all four universities, stratified by nationality.

### Self-reported vaccination status of medical students at German and Hungarian places of study

A complete pertussis vaccination status was reported by 69.8% of the medical students in Dresden, the highest percentage reported from any of the four universities (56.8% Munich, 40.5% Pécs, 42.2% Budapest). In Dresden, the amount of students with a complete vaccination status was significantly higher among Germans than students of other nationalities (70.8% vs 50.0%, *p* ≤ 0.05). In Pécs, only 25.1% of the students of other nationalities reported to have a complete vaccination status.

The percentage of students with self-reported basic immunization against pertussis was highest in Budapest (35.3%). At both Hungarian universities, considerably more Hungarians than others reported to have basic immunization (s. Table 2). A higher amount of students in Munich reported no pertussis vaccination (6.0% vs 1.4% Dresden/ 2.7% Pécs/ 1.1% Budapest, *p* ≤ 0.05). In Budapest, more German students than others reported not being vaccinated (6.0% vs 0.5% Hungarian and 0.0% other, *p* ≤ 0.05). The percentage of students not knowing their pertussis vaccination status was highest in Pécs (28.3% vs 16.2% Dresden/ 20.0% Munich/ 21.4% Budapest, *p* ≤ 0.05). In all study sites more students from other countries than Germany and Hungary were unsure about their vaccination status (28.6–32.2% vs. 47.5% in Pécs; *p* ≤ 0.05).

### Self-reported vaccination status of German medical students’ subgroup in Germany and Hungary

German medical students at the universities in Germany (*n* = 920) and Hungary (*n* = 297) showed significant differences in their self-reported vaccination status (χ^2^ = 37.6/ df = 3/ *p* ≤ 0.001). A significantly higher percentage of German students in Germany stated a complete vaccination status (65.1% vs 46.1%, *p* ≤ 0.05). German students in Hungary reported to have basic immunization more often than German students in Germany (19.5% vs 14.6%, *p* ≤ 0.05). This group (German students in Hungary) also reported uncertainty about their vaccination status more often than German students in Germany (30.3% vs 16.7%, *p* ≤ 0.05). There was no significant difference between the students with regard to the status of not being vaccinated against pertussis at all.

### Self-reported vaccination status according to year of study

Students at different stages of their studies showed significant differences (*p* ≤ 0.05) in their pertussis vaccination status. The number of students with self-reported complete vaccination status increased in accordance with the year of study (40.9% first year vs 53.5% third year vs 62.0% fifth year). Dependent on study place, the association between study year and complete vaccination status differ: there were significant increases of proportions of students reporting complete status correlated to increasing study years (1st-3rd-5th) in Dresden (*p* ≤ 0.05). In Budapest and Munich there were only significant differences of these proportions between the first and the third/fifth study year (*p* ≤ 0.05). In Pécs, there were no significant differences of the proportions dependent on study years. More students in their third or fifth year than first-year students reported to have basic immunization (28.4 and 30.6% vs 20.7%). The percentage of students not being vaccinated against pertussis is higher in the first year than in the fifth year (3.5% vs 1.1%). The number of students not knowing their vaccination status decreases in accordance with the year of study (34.9% first year vs 16.1% third year vs 6.3% fifth year).

### Subjective importance assessment of pertussis vaccination

The question about the importance of the vaccination against pertussis was answered by 2592 medical students (92.0%). 91.2% of these students indicated their nationality (*n* = 2569). Table 3 shows the results stratified by place of study and nationality.

**Table 3:** Assessed importance of pertussis vaccination by university and nationality among medical students, *n* = 2569

		Assessed importance of pertussis vaccination by university and nationality; % (n)			
University	Nationality (n)	Absolutely necessary	Partly necessary	Mostly unnecessary	Unnecessary/ dangerous
Dresden	German (*n* = 527)	77.4%_a_ (408)	19.2%_a_ (101)	2.8%_a_ (15)	0.6%_a_ (3)
	Hungarian (*n* = 0)	0.0% (0)	0.0% (0)	0.0% (0)	0.0% (0)
	Other (*n* = 29)	58.6%_b_ (17)	31.0%_b_ (9)	10.3%_b_ (3)	0.0%_a_ (0)
	All (*n* = 556)	76.4% (425)	19.8% (110)	3.2% (18)	0.5% (3)
Munich	German (*n* = 364)	63.7%_a_ (232)	26.9%_a_ (98)	7.1%_a_ (26)	2.2%_a_ (8)
	Hungarian (*n* = 0)	0.0% (0)	0.0% (0)	0.0% (0)	0.0% (0)
	Other (*n* = 52)	57.7%_a_ (30)	34.6%_a_ (18)	5.8%_a_ (3)	1.9%_a_ (1)
	All (*n* = 416)	63.0% (262)	27.9% (116)	7.0% (29)	2.2% (9)
Pécs	German (*n* = 181)	61.9%_a_ (112)	27.6%_a_ (50)	7.7%_a_ (14)	2.8%_a_ (5)
	Hungarian (*n* = 378)	85.7%_b_ (324)	11.9%_b_ (45)	2.1%_b_ (8)	0.3%_b_ (1)
	Other (*n* = 174)	60.9%_a_ (106)	26.4%_a_ (46)	10.3%_a_ (18)	2.3%_a_ (4)
	All (*n* = 733)	73.9% (542)	19.2% (141)	5.5% (40)	1.4% (10)
Budapest	German (*n* = 108)	66.7%_a_ (72)	22.2%_a_ (24)	10.2%_a_ (11)	0.9%_a_ (1)
	Hungarian (*n* = 560)	86.6%_b_ (485)	10.4%_b_ (58)	2.7%_b_ (15)	0.4%_a_ (2)
	Other (*n* = 196)	74.0%_a_ (145)	21.4%_a_ (42)	3.1%_b_ (6)	1.5%_a_ (3)
	All (*n* = 870)	81.3% (702)	14.4% (124)	3.7% (32)	0.7% (6)
Total	German (*n* = 1180)	69.8%_a_ (824)	23.1%_a_ (273)	5.6%_a_ (66)	1.4%_a_ (17)
	Hungarian (*n* = 938)	86.2%_b_ (809)	11.0%_b_(103)	2.5%_b_ (23)	0.3%_b_ (3)
	Other (*n* = 451)	66.1%_a_ (298)	25.5%_a_ (115)	6.7%_a_ (30)	1.8%_a_ (8)
	All (*n* = 2569)	75.2% (1931)	19.1% (491)	4.6% (119)	1.1% (28)

_a,b_ Each letter specifies a subset of ‘Nationality’ categories whose column shares are not significantly different at the 0.05 level (Z-test)

75.2% of all the students assessed the pertussis vaccination as absolutely necessary, with the highest percentage among Hungarian students (86.2%, *p* ≤ 0.05). This assessment was given by more students in Budapest than in Pécs and Munich (81.3% vs 73.9% vs 62.9%, *p* ≤ 0.05). In Dresden, a higher number of German students, compared to students from other countries, assessed the pertussis vaccination to be absolutely necessary (77.4% vs 58.6%, *p* ≤ 0.05). At the Hungarian universities, this assessment was given by more Hungarian than international students (Table 3). 19.0% of all students assessed the pertussis vaccination as partly necessary, among those the Hungarian students with the lowest percentage (11.0% vs 23.1% German and 25.5% other, *p* ≤ 0.05). In Munich, the number of students assessing the vaccination as partly necessary was considerably higher than at the other three universities (27.7% Munich vs 19.6% Dresden/ 19.1% Pécs/ 14.4% Budapest). 4.7% of the students considered the pertussis vaccination to be mostly unnecessary, 1.1% unnecessary/dangerous.

### Importance assessment of pertussis vaccination among German students’ subgroup in Germany and Hungary

German students’ statements in Germany (*n* = 891) and Hungary (*n* = 289) showed significant differences in the importance assessment of the pertussis vaccination ((χ^2^ = 10.56/ df = 3/ *p* ≤ 0.014): German students in Germany assessed the vaccination as being absolutely necessary more often than German students in Hungary (71.8% vs 63.7%, *p* ≤ 0.05), while more German students in Hungary assessed the vaccination as being mostly unnecessary (8.7% vs 4.6%, *p* ≤ 0.05).

### Importance assessment of pertussis vaccination according to year of study

The statements about the importance of the pertussis vaccination in the first, third and fifth year of study show the following differences (χ^2^ = 59.36/ df = 9/ *p* ≤ 0.001): The percentage of students assessing the vaccination as absolutely necessary increased in accordance with the years of study (64.7% first year vs 77.6% third year vs 90.0% fifth year, *p* ≤ 0.05). At the same time, there was a decrease in the percentage of students assessing the vaccination as partly necessary (26.3% first year vs 18.6% third year vs 7.2% fifth year, *p* ≤ 0.05) or mostly unnecessary (7.2% first year vs 2.2% fifth year, *p* ≤ 0.05) during the course of their studies. Also, the percentage of students evaluating the vaccination as unnecessary/dangerous decreased as they progressed in their studies, though insignificantly.

### Factors influencing a self-reported complete vaccination status

Bivariate analyses showed significant associations between place of study, nationality, year of study, subjective assessment and the self-reported pertussis vaccination status. In order to weight these factors and analyze their level of prediction on the self-reported complete vaccination status, they were merged in a multivariate regression model.

The results of the logistic regression (Nagelkerke R^2^ 0.136) showed significant effects of these factors: place of study (university), nationality and the subjective assessment of the pertussis vaccination being absolutely necessary (Table 4). Medical students in Dresden and Munich were more likely to be completely immunized (OR 2.95, *p* ≤ .001 und OR 1.72, *p* = .004) than those in Budapest. Also, the nationality predicted the vaccination status: Students from Hungary or other nations had a lower likelihood of a self-reported complete vaccination status (OR 0.52, *p* ≤ .001 and OR 0.63, *p* = .008). Those assessing the vaccination against pertussis as absolutely necessary reported 2.8 times more often to be completely immunized than those not assessing the vaccination as absolutely necessary.

**Table 4:** Predictors of complete (basic and booster) pertussis vaccination among medical students

	Odds ratios^a^	95% confidence intervals
University		
Budapest (ref.)	1	
Dresden	2.95*	2.02–4.32
Munich	*1.72* ^†^	1.19–2.49
Pécs	1.09	0.87–1.39
Nationality		
German (ref.)	1	
Hungarian	0.52*	0.38–0.71
Other	*0.63* ^†^	0.45–0.89
Study year		
1 (ref.)	1	
3	0.98	0.78–1.24
5	1.07	0.83–1.37
Pertussis vaccination assessed as “absolutely necessary”		
no (ref.)	1	
yes	2.79*	2.16–3.61

^a^Odds ratios adjusted for university, nationality, study year and pertussis vaccination assessed as ‘absolutely necessary’

*Levels of significance: *p* ≤ .001

^†^Level of significance: *p* ≤ .01

## Discussion

Only half of the participating medical students reported a complete pertussis vaccination status, i.e. they reported to have received both the basic immunization and booster vaccination. Based on the study results, differences in the vaccination statements were associated with the nationality: The number of participants reporting a complete vaccination status was highest among German medical students, in comparison to students from Hungary and other nations. Furthermore, studying at a German university was positively associated with a complete vaccination status. Nevertheless, based on the differences in vaccination policies in Germany and Hungary, these results must be discussed country specific.

Our data on pertussis vaccinations among German students showed comparably better and yet insufficient vaccination rates (60.5%). According to earlier studies from Germany and Switzerland based on self-reported data, 58.0% of medical students in Frankfurt [19] and 42.7% of students at the pediatric clinic at Basel University [20] reported to have received the pertussis vaccine in the past 10 years. Another study from Frankfurt reported even lower figures: Only 17.1% of the medical students reported a vaccination against pertussis in the past 10 years [23]. Another smaller study (*n* = 82) with vaccination certificates identified comparable vaccination rates (53,7%) regarding complete pertussis vaccination among German nursing students, as in our study [28]. These results together with the high number of pertussis cases per year imply a suboptimal pertussis immunization ratio among people working in healthcare context in Germany.

We observed regional differences in vaccination status due to the study site in Germany. The percentage of students with self-reported booster vaccination was higher in Dresden than in Munich. Also, Dresden students estimated the pertussis vaccination as absolutely necessary more often than students in Munich. Possibly, the effects of the divided Germany with their different vaccination regulations in the past are still noticeable: other than in the former western Germany, in the German Democratic Republic (GDR) many vaccinations (e.g. pertussis) were mandatory. This could still have an impact on today’s students’ vaccination behavior in Dresden, which is a city on former GDR territory. These regional differences have been analyzed also in a study on adults’ health in Germany (DEGS1), showing that 22.9% of the women and 20.3% of the men in Eastern Germany were vaccinated against pertussis in the past 10 years, while only 11.8% (women) and 9.8% (men) received the vaccination in the Western part [29].

Our data regarding low vaccination rates among Hungarian students, however, has to be discussed critically. Vaccination against pertussis is mandatory for children in Hungary and 99.6% of Hungarian children have received booster vaccinations [30]. In addition, there are very few reported pertussis cases per year [4]. Relaying on this evidence, Hungarian students were expected to report a better vaccination status than Germans. This was not confirmed by our study, where a major part of Hungarian students reported to only have received basic immunization. Nevertheless, when summing up the rates for basic immunization with and without booster, the percentage of students reporting any pertussis vaccination is highest among the Hungarians (82.6% vs 76.3% German, 60.3% others). This implies that most Hungarian students do not know their own vaccination status very well, although only 16.5% reported not knowing their status. Due to the mandatory vaccination program students are most likely still sufficiently vaccinated in their first study years, but low awareness of their vaccination status could have implications for future booster vaccinations.

The incomparability of data regarding Hungarian students could be explained by the point of time for the last booster: according to current recommendations this mandatory vaccination takes place on the 6th grade at school [9]. Nevertheless, students in our study got their booster at the age of 6 years which might not be considered as a booster vaccination. Further, getting a booster every 10 years was not yet recommended in Hungary at the time of the study. Another explanation could be the Hungarian vaccination policy (based on mandatory vaccinations), giving a sense one does not need to actively care about one’s vaccination status.

All in all, more than one fifth of all students indicated not knowing their own pertussis vaccination status. Among future doctors, this is a surprisingly large proportion, though smaller compared to other studies (Petersen et al.: 33.5% and Wicker & Rose: 36.6%) [19, 23]. More students in the international cohort (other than German, Hungarian) reported to not know their own vaccination status (37.4%) and only one third reported a complete vaccination status. In contrast, German and Hungarian students studying in their home countries reported lower rates (15.5–18.4%) for not knowing their vaccination status. In Pécs in particular, where nearly half of students have a non-Hungarian nationality, many students admitted not knowing their vaccination status. Based on the increasing migration but also the growing number of medical students moving abroad for and during their studies, students with insufficient vaccination status have a higher risk to pass an infection among colleagues or patients or when travelling in countries with higher incidence (e.g. Germany). Control of vaccination status and boosters should be offered especially for the international students.

To analyze the effects of studying abroad on vaccination status, we compared data on German students in Germany with those studying in Hungary. It was expected that the students moving to Hungary would be more likely to have complete immunization, as a preparation for their stay abroad. However, this was not confirmed by our study: German students in Germany reported more often to be completely vaccinated against pertussis than those in Hungary. Also, a higher number of the students in Germany gave particulars about their vaccination status and assessed the pertussis vaccination as absolutely necessary, according to their self-reported – better – vaccination status. In this case, studying abroad did not have any positive effects on the knowledge or the status of the vaccination. Nevertheless, the reasons could not be explained by the current data.

Further, our bivariate analysis in the whole sample showed that the students’ vaccination status differ according to the academic year they are in. As expected, the number of students with a complete vaccination status did increase from year one to year five. But the association differs dependent on study place: more differences between study years were observed at the German universities and Budapest; no association was observed in Pécs. Within the multivariate analysis (logistic regression) the influence of the study year on the reported complete vaccination status was not confirmed. This might be explained by different (partly no or weak) associations at several study places. The influence of nationality and study place on the complete vaccinations status was significant and even stronger.

The knowledge about the personal vaccination status and the number of those assessing the pertussis vaccination as absolutely necessary increased as well. Positive effects on the vaccination status and the subjective assessment could be due to increasing knowledge of vaccinations and contact with occupational medical consulting services /student health services during the studies.

Informing students is important, since only 60.8% of medical students have knowledge of the general pertussis vaccination recommendation for medical staff in Germany, as reported by Petersen et al. [19].

In general, the survey about the importance assessment of the pertussis vaccination revealed that three out of four participating medical students assessed the vaccination as absolutely necessary. In the total sample, more than 91% of all student groups (and 97% of Hungarians) considered vaccinations partly/absolutely necessary. Our multivariable results offered that students who assess pertussis vaccination as absolutely necessary have higher chances to be complete vaccinated against pertussis that is comparable to other investigations [31, 32].

Due to the positive attitudes towards vaccination, there is potential to sensitize medical students for the relevance of a complete vaccination status and improve vaccination status by offering low-threshold consulting and vaccination in frames of the in-house medical services in the university setting. There is evidence that the vaccination figures improve, if medical students participate in health checks including certificate checks and vaccination recommendations prior to clinical work [33]. The problem in Germany is that vaccinations indicated through occupational medical offices are often not available at medical faculties [34] and student health services do not exist in all universities: students have to take care of the control and update of their vaccinations outside the university setting.

### Limitations

Since data on vaccination status was based on self-report (without a vaccination certificate), the recall and response bias are possible. Validity and reliability of surveys involving self-reported information can vary in quality. Self-reported information about one’s vaccination status are not necessary sufficient to gain a reliable picture of the reality [35] but they rather show tendencies and give valuable information regarding the awareness of one’s vaccination status. Nevertheless, the self-reported data on vaccination status of German students was comparable with data based on vaccination records among health professionals in another German study [28]. To gain more exact data on vaccination status, future studies should include a control of vaccination certificate. Further, serological tests to collect data on vaccination status are cost-intensive and not feasible within the scope of our multicenter study about medical students’ health behavior in a large international student population.

A further bias could have caused by perception of the term “booster vaccination”. According to both German and Hungarian vaccination regulations, vaccinations scheduled for children from age 5–6 (Germany) and 18 months (Hungary) already are called “boosters” [7, 9]. In some other countries, the pertussis vaccinations in child age are given in frames of the routine/primary infant schedule and vaccination is called as “booster” somewhat later, from age 8–12 years. Students might have confused “basic immunizations” with the concept of “booster” and thus, they might have underestimated their pertussis vaccination status.

## Conclusions

The results of this study showed that medical students, regardless of their origin, had very positive attitudes towards vaccinations. However, our study identified a large group of medical students with insufficient vaccination and of students that are not aware of their vaccination status. Based on our study, a special focus should be put on international students. Although medical students show a better vaccination status and acceptance level than students from other faculties [26] they should be in particular sensitized for the relevance of vaccination. This is important since a complete vaccination status is elementary to protect own, patient and public health, especially for people working in healthcare. Further, being aware of one’s vaccination status is elementary for following the current vaccination recommendations (also outside the mandatory vaccination programs) and taking action to get a pertussis booster vaccination every 10 years.

Multilingual on-campus campaigns, in-house medical services and education on infectious diseases and how to prevent them could help to improve knowledge and vaccination status, especially for potential risk groups, such as students with only basic immunization or with no knowledge about their vaccination status. Particularly, free vaccinations during occupational medical check-ups have the potential to lower the vaccination threshold, reduce distances, and efficiently improve vaccination numbers.

## Abbreviations

dTap: Combined vaccine against diphtheria, tetanus and pertussis with acellular pertussis component (booster)
EEA: European Economic Area
OR: Odds ratio
SD: Standard Deviation
SF-36: 36-Item Short Form Survey
WHO: World Health Organization
